# The effects of microgravity on bone structure and function

**DOI:** 10.1038/s41526-022-00194-8

**Published:** 2022-04-05

**Authors:** Joey Man, Taylor Graham, Georgina Squires-Donelly, Andrew L. Laslett

**Affiliations:** 1 Commonwealth Scientific and Industrial Research Organisation (CSIRO) Manufacturing, Clayton, Victoria 3168 Australia; 2 Australian Regenerative Medicine Institute, Monash University, Melbourne, Victoria 3800 Australia; 3 Space Technology Future Science Platform, Commonwealth Scientific and Industrial Research Organisation (CSIRO), Clayton, Victoria 3168 Australia

**Keywords:** Cell biology, Biotechnology

## Abstract

Humans are spending an increasing amount of time in space, where exposure to conditions of microgravity causes 1–2% bone loss per month in astronauts. Through data collected from astronauts, as well as animal and cellular experiments conducted in space, it is evident that microgravity induces skeletal deconditioning in weight-bearing bones. This review identifies contentions in current literature describing the effect of microgravity on non-weight-bearing bones, different bone compartments, as well as the skeletal recovery process in human and animal spaceflight data. Experiments in space are not readily available, and experimental designs are often limited due to logistical and technical reasons. This review introduces a plethora of on-ground research that elucidate the intricate process of bone loss, utilising technology that simulates microgravity. Observations from these studies are largely congruent to data obtained from spaceflight experiments, while offering more insights behind the molecular mechanisms leading to microgravity-induced bone loss. These insights are discussed herein, as well as how that knowledge has contributed to studies of current therapeutic agents. This review also points out discrepancies in existing data, highlighting knowledge gaps in our current understanding. Further dissection of the exact mechanisms of microgravity-induced bone loss will enable the development of more effective preventative and therapeutic measures to protect against bone loss, both in space and possibly on ground.

## Introduction

Extended human spaceflight was once a distant fantasy; however, it is now almost a tangible reality. With NASA’s goal to send humans back to the moon by 2024, then onwards to Mars in the 2030s, it is now more critical than ever to understand the impacts of long-term space travel on human health^1^. Among the many technical, logistical and physiological challenges inherent to extended space exploration, the loss of gravitational force is a major prohibitive environmental factor that adversely affects the body of space travellers. The human body is intrinsically adapted to Earth’s gravity (~9.907 m/s^2^), thus exposure to conditions of reduced gravity, or microgravity (µG) can lead to a plethora of complications in normal bodily functions. µG decreases the effort required for movement, while causing mass fluid redistribution^2^. As a result, muscles in the arms and legs experience atrophy^3^, the cardiovascular system is compromised^4^, the immune system is suppressed^5^, and increased cranial pressure leads to vision problems and neurological impairments^6^. Exposure to µG also results in skeletal deconditioning, where significant reductions in bone mass increases the risk of fractures and osteoporosis, threatening the viability of long-duration missions and astronauts’ mobility upon return to Earth^7,8^.

### A brief background to bone homeostasis

**Fig. 1:**
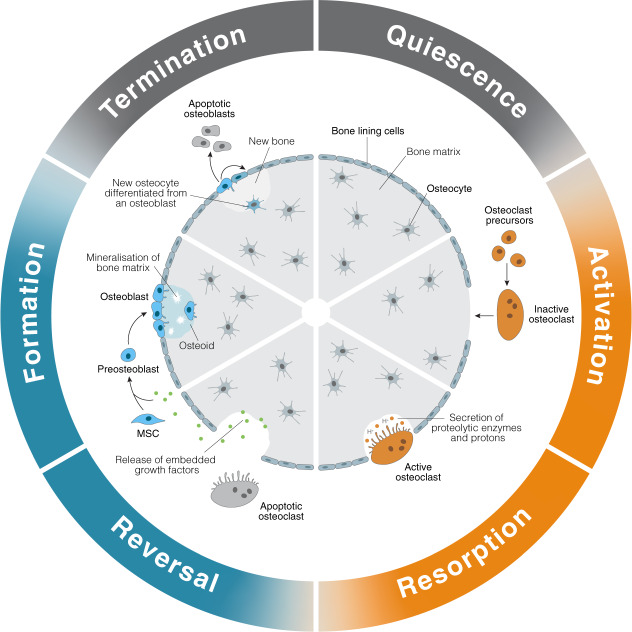
The stages of bone remodelling. Bone remodelling is a process where cycles of bone resorption and formation are separated by periods of quiescence. During quiescence, the relatively inactive bone surface is lined by flat remnants of osteoblasts. Events such as hormone detection and/or mechanical loading can activate the recruitment of circulating osteoclast precursor cells. These precursor cells fuse to form premature osteoclasts and migrate to the bone surface, while bone lining cells retract to enable preosteoclast binding. Once bound to the bone matrix to form a sealing zone in the isolated area, they differentiate into mature osteoclasts for bone resorption. Mature osteoclasts secrete protons to create an acidic environment that dissolves bone mineral, and proteolytic enzymes to digest the bone matrix. The resorption process results in the formation of cavities, also known as Howship’s lacunae, beneath active osteoclasts. Osteoclasts undergo apoptosis once these cavities reach a certain size, leading to the termination of bone resorption. The bone degradation process also releases embedded growth factors that reverses bone resorption by recruiting and stimulating the differentiation of mesenchymal stem cells (MSCs) into bone-forming osteoblast lineage cells. Once recruited to the lacunae, preosteoblasts secrete a variety of matrix proteins in the organic bone matrix, or the osteoid, which are then mineralised by mature osteoblasts. Bone formation is terminated upon completion of mineralisation. Osteoblasts either undergo apoptosis or differentiation into quiescent bone lining cells. Alternatively, osteoblasts can become embedded in the bone matrix to form osteocytes, which form a canalicular network of branched dendritic processes to communicate with bone lining cells, osteoblasts, and other osteocytes.

Before examining how bone is affected by microgravity, it is important to understand bone function in 1G. Under normal circumstances, bone remodeling is an adaptive and balanced process where bone resorption and formation are coupled to regulate homeostasis of bone tissue^9^. The overall process relies on osteoblasts and osteoclasts acting in concert to regulate bone formation and resorption, respectively. The inactive bone surface is lined with flat remnants of osteoblasts, where they serve as a membrane capable of detecting hormones and/or mechanical loading to initiate the bone remodeling process^9–13^ (Fig. 1). Once circulating osteoclast precursors are recruited to sites of bone remodeling, they differentiate into mature osteoclasts that secrete enzymes such as cathepsin K and metalloproteinase to digest the collagen-rich bone matrix^14^. This degradation process also releases calcium and embedded growth factors, such as bone morphogenic proteins (BMPs) and transforming growth factor-β (TGFβ), which contribute to bone formation^9,13^. Once the cavities beneath resorbing osteoclasts reach a certain size, osteoclasts undergo apoptosis to terminate bone resorption and prevent excess bone loss^15^.

The newly liberated growth factors from bone degradation can recruit and stimulate the differentiation of mesenchymal stem cells (MSCs) to osteoblast lineage cells, including osteoprogenitors, osteoblasts and osteocytes^16^. Maturing osteoprogenitors and preosteoblasts secrete a variety of matrix proteins, such as type 1 collagen, as well as non-collagen proteins (osteocalcin, osteonectin, bone sialoprotein II and osteopontin) and proteoglycans, which are mineralised by mature osteoblasts^17^. In addition, preosteoblasts express alkaline phosphatase (ALP) for bone mineralisation, hence its expression and activity are key markers of osteoblast differentiation and maturation^18,19^. Osteoblasts either undergo apoptosis, differentiate into quiescent bone lining cells, or become embedded in the bone matrix to form osteocytes, which form a canalicular network of branched dendritic processes^20,21^. They communicate with bone lining cells, osteoblasts and other osteocytes, and are suggested to influence bone remodeling in response to mechanical loading^17,22^. Thus, with an understanding of bone homeostasis, what is currently known regarding the effect of microgravity on bone will be reviewed below.

## The effect of microgravity on bone

### Microgravity-induced bone loss in humans

The first observation of µG-induced bone loss was recorded in the mid-1970s, when Skylab crew members demonstrated the loss of 1–2% bone mass per month compared to pre-flight and ground controls^23–25^. Since then, despite the implementation of preventative exercises, bone loss in space has been one of the most frequently observed outcomes among astronauts (Table 1, row 1-7). The weightlessness experienced in microgravity reduces the loading on weight-bearing bones, resulting in adaptive changes that increase bone resorption and inhibit bone formation^26^. Indeed, bone mineral density (BMD) studies of astronauts demonstrate substantial decrease in the mass of weight-bearing bones such as the tibia, but not in non-weight-bearing bones like the distal radius^8,27–29^. Bone resorption is particularly exacerbated in the first 2 weeks of spaceflight, where urinary concentrations of resorption markers such as N-telopeptide and pyridinium crosslinks are increased^26,30–33^. On the other hand, urinary calcium levels are increased^24,32,34^, indicating reduced calcium absorption in astronauts^32,34^. As bone formation is reportedly unchanged or decreased^30,32,33^, this results in an overall negative calcium balance that contributes to bone loss in space. Notably, a recent study suggests that circulating biomarkers of bone turnover pre-flight can predict the severity of in-flight bone loss, where astronauts with elevated bone resorption and formation markers pre-mission experience greater losses in BMD and strength of their distal tibia during spaceflight^29^.

**Table 1:** Spaceflight studies of bone-loss in humans.

Species	Duration (days)	Sample size	µG-related observations	Reference
Human	126–438	45	• Spaceflight decreases BMD • Recovery to pre-flight BMD takes longer than flight duration	Sibonga et al.^28^
Human	28, 183	2	• Decreased bone mass in weight-bearing tibia but not non-weight-bearing distal radius • Bone loss more severe in longer spaceflight • Recovery takes longer than flight duration	Collet et al.^27^
Human	115	3	• Bone formation markers (ALP and osteocalcin) decreased during spaceflight • Calcium metabolism disturbed during spaceflight – calcium excretion increased while intake and absorption decreased	Smith et al.^70^
Human	180	4	• Bone formation depressed with decreased markers (ALP, collagen type 1 and osteocalcin)	Caillot-Augusseau et al.^26^
Human	60–460	15	• BMD reduced in weight-bearing tibia but not non-weight-bearing distal radius	Vico et al.^8^
Human	181	8	• Total BMD of spine, femur, hip and femoral neck decreased during spaceflight • Bone loss and recovery rate differs between trabecular and cortical compartments	Dana Carpenter^148^
Human	121–182	14	• BMD of spine and hip reduced at the rate of 0.9%/month and 1.4–1.5%/month, respectively • All integral, cortical and trabecular compartments are affected, although more severe in the hip than the spine	Lang et al.^149^

Summary of bone-related observations in humans from various studies during spaceflights compared to respective ground controls.

*BMD* bone mineral density, *ALP* alkaline phosphatase.

The severity of bone loss also increases with spaceflight duration, and the time required for recovery to pre-flight BMD levels is reportedly longer than the actual mission^27,28^. Another study evaluates the bone mass, microarchitecture and strength of 13 astronauts who spent 4–6 months aboard the International Space Station (ISS)^35^. This study monitored skeletal recovery of each astronaut for up to 12 months post-landing. Although the cortical bone thickness and density of the weight-bearing distal tibia eventually recover upon landing, the cortical porosity and trabecular bone fail to recover, leading to reduction in the ultimate load of the bone^35^. Congruent to previous findings^8,36,37^, the non-weight-bearing distal radius is preserved at landing^35^. Interestingly, this study suggests that the distal radius suffers progressive fragility 6 months after landing, which coincided with bone remodeling markers declining to below pre-flight levels between 6 and 12 months upon return^35^. The observation of progressive fragility in non-weight-bearing bones might have escaped other studies due to inadequate length of recovery monitoring. Future studies should increase follow-up duration to validate this phenomenon.

### Microgravity-induced bone loss in animals

Despite the physiological relevance, relying solely on astronaut data to understand µG-induced bone loss is limiting for many reasons. One of which is the rarity of human spaceflight and the limited number of astronauts per mission, thus leading to the challenge of small sample sizes in these studies. The smaller physical sizes of animals such as rats, mice and fish allow for more compact storage in space missions, enabling larger sample sizes while maintaining some physiological relevance. Similar to observations in astronauts (Table 1), µG exposure for 8 days decreases overall bone volume and thickness by 6.3% and 11.9%, respectively, in 15 mice^38^. The negative effects of µG on trabecular bone mass are also reportedly discernible in weight-bearing bones such as tibia, femur and vertebrae^39^, complementing observations in human studies. Seven rats aboard the Soviet mission COSMOS 1667 for 7 days demonstrate a reduction in tibial trabecular bone volume by 47–55%, trabecular thickness by 20–24% and density by 40–43% compared to ground controls^36^. In addition, another study observes a 64% decrease in femoral trabecular bone volume and a 140% increase in bone resorption in 16 mice following 30 days of spaceflight compared to control mice on Earth^40^. Congruently, a larger sample size of 40 mice subjected to 22 days of µG exposure exhibit significant reductions in BMD of both left and right femur compared to ground controls, albeit to a smaller extent of 11% and 8% respectively^41^.

On the contrary, there is opposing evidence that suggests µG-induced bone loss may not be strictly related to its weight-bearing nature^41^. The aforementioned study also reports that BMD of the humerus, a weight-bearing bone, is not affected in spaceflight mice compared to ground controls despite changes in both femurs^41^. Similarly, 14 days of spaceflight reduces the BMD of femurs, but has no effect in the humerus of 12 rats compared to controls on Earth^42^. It should be noted that these rats are ovariectomized^42^, which amplifies phenotypes of bone loss as ovarian hormones are crucial regulators of skeletal growth^43^. The relationship between µG and the weight-bearing nature of bone loss remains unclear given the contradicting evidence presented. Nevertheless, these studies collectively highlight the damaging effects of µG on bone health during space travel, as well as the risk of premature osteoporosis from the uncertainties of post-flight skeletal recovery.

### Limitations of studying bone loss in space

Spaceflight experiments are certainly the most physiologically relevant method of studying µG-induced bone loss, however, they come with significant limitations. Firstly, mission launches are infrequent, hence opportunities for experiments in space are limited. Secondly, experimental design must comply with cargo weight and space constraints in both the launch and space station. This often leads to small sample sizes and minimal, if any, biological replicates, which reduces statistical power and rigour of the research. Thirdly, the engineering components must be sound, ensuring that the experimental model survives the harsh launch conditions, the duration of data collection in orbit, and in some cases, the return to Earth. The strategies to overcome or bypass these restrictions ultimately feed into the final limitation – cost. Experiments requiring astronaut intervention incur even higher financial burdens, hence simplistic autonomous or remotely controlled experiments are favoured. In light of these limitations, many researchers elect to study µG-induced bone loss using simulated µG here on Earth.

## The effect of simulated microgravity on bone

### Current microgravity simulation technologies

To address the logistical problem of studying bone loss directly from humans in space, several ground-based methods have been developed since the 1970s to subject various models, such as cells, plants, animals and humans, to near-µG conditions. The following discussion will be limited to technologies relevant to research on µG-induced bone loss (Table 2); a wider scope of methods is thoroughly reviewed by Ferranti et al.^44^.

**Table 2:** Advantages and disadvantages of microgravity simulation techniques.

Technique	Advantages	Disadvantages	Reference
HBR/HDT (human)	• Fluid redistribution similar to µG • Robust body of work • Enables examination of multiple body systems • Suitable for long-term studies	• Gravitational force on bodyweight is not lost • Compression of skin surface against bed does not occur in true µG • Seven-fold reduction in microgravitational effect compared to dry immersion	Tomilovskaya et al.^53^
HLU (rodents)	• Fluid redistribution similar to µG • Robust body of work • Enables examination of multiple body systems • Suitable for long-term studies	• Internal organs still subjected to gravity	Tesch et al.^54^
Water immersion (human)	• Robust body of work • Enables examination of multiple body systems • Useful in astronaut training	• Imbalance of µG across limbs • Side effects on osmotic balance from immersion • Not suitable for long-term studies	Duddy et al.^50^ Tsai and Maibach^51^
Dry immersion (human)	• Robust body of work • Enables examination of multiple body systems • Useful in astronaut training	• Imbalance of µG across limbs	Shulzhenko et al.^52^ Tomilovskaya et al.^53^
RWV (cells)	• Robust body of studies • Well-established method • Near true µG • Varying rotation speeds available	• Susceptible to shear forces and vibration • Potential centrifugal force on samples distant from rotation axis • Formation of multicellular spheroids/cell aggregates • Fast rotating clinostats cannot house large or many samples • For long-term studies, media change requires pausing rotation • Not applicable for examining acute responses to µG	Brungs et al.^59^ Krause et al.^60^ Svejgaard et al.^62^ Loon^63^ Wuest et al.^64^
RPM (cells)	• Robust body of studies – but less than RCC/RWV • Near true µG • Programmable to simulate gravity of any planet	• Susceptible to shear forces and vibration • Samples further away from centre stage experience centrifugal force • Tendency for multicellular aggregate formation in non-adherent cell samples • Pause in rotation required for media change in long-term studies • Not applicable for examining acute responses to µG	Brungs et al.^59^ Krause et al.^60^ Svejgaard et al.^62^ Loon^63^ Wuest et al.^64^
Freefall machine (cells)	• Designed to measure acute responses to µG	• Short window of µG • Adverse effects during hyper-gravity window in between falls • Small body of work	Schwarzenberg et al.^57^ Mesland et al.^56^

This is not an exhaustive list of techniques available, only microgravity simulators relevant to bone-loss experiments are discussed here.

*HBR* horizontal bed rest, *HDT* head-down tilt, *µG* microgravity, *HLU* hindlimb unloading, *RWV* rotating wall vessels, *RPM* random positioning machine.

#### Horizontal bed rest/head-down tilt

Initially, horizontal bed rest (HBR) was used to mimic the inactivity of the human body in weightlessness. However, this method failed to recapitulate fluid redistribution towards the head as observed in inflight astronauts. As such, researchers experimented with tilting the subjects towards the head to encourage cephalad redistribution from the legs^45–47^. Head-down tilt (HDT) angles range from 4° to 15°, but a tilt angle of 6° which approximates to 0.1 G became the analogue for µG simulation in most human bed rest studies^48^. Comparisons between HBR/HDT and µG are thoroughly discussed by Hargens et al.^49^.

#### Water immersion

While HBR/HDT simulates microgravity by minimising G forces, water immersion achieves microgravity via neutral buoyancy. Neutral buoyancy describes the displacement of a medium which a mass is immersed in, resulting in the balance of gravitational force. During water immersion, the subject usually sits in water with a temperature of 34–35 °C. Such facilities have been utilised since the 1960s to prepare astronauts for spaceflight, as well as in experiments involving µG simulation^50^. However, immersing subjects in water for prolonged periods can lead to adverse cutaneous effects^51^. As such, the technique of dry immersion was introduced.

#### Dry immersion

In keeping with the neutral buoyancy concept of water immersion, subjects are immersed in a bath to neck level, but are “kept dry by the use of a waterproof, highly elastic cloth”^52^. While some localised pressure can be experienced at the seat and/or feet during the conventional water immersion, the buoyant force from the air between the elastic cloth and skin in dry immersion effectively prevents any localised surface pressures. Furthermore, dry immersion enables longer experiments for up to 56 days, while reproducing µG-induced physiological effects similar to HDT experiments^53^. The history, utilisation and effects of dry immersion is extensively reviewed by Tomilovskaya et al.^53^.

#### Rodent HLU

The hindlimb unloading (HLU) model is the most common method to simulate spaceflight conditions in rodents, where rats or mice are suspended by their tails, or the use of surgical pins or body harnesses^54^. This causes the removal of mechanical loading from the hindlimbs, as well as head-ward fluid shifts comparable to the human HDT model^55^. Despite the hindlimb suspension, it should be noted that internal organs remain affected by gravity, thus HLU presents as a limited model for µG.

There are 2 common models of replicating µG in research involving small biological samples like cell cultures: free fall state and clinorotation. When cells reach terminal velocity during a state of free fall, they are unable to respond to gravitational force. As such, the cells are in a state of functional weightlessness. The Free Fall Machine (FFM) involves dropping a sample in a long vacuum tube, resulting in a free fall for 900 milliseconds (ms) before being propelled back to the top by a current of air at ~2–20 times normal gravity for 80 ms^56^. This theory relies on the assumption that cells do not have sufficient time to react to the small hyper-gravity windows between free falls^56^, which was later disproved^57,58^.

Clinorotation models µG by rotating a sample with enough speed to disable adaptation of gravity vector, but slow enough to prevent generation of centrifugal shear forces. Depending on the number of rotation axes, clinostats can be classified into 2 major classes:

#### 1D/2D Clinostat – RWV

1D clinostats rotate the specimen along its vertical axis, while 2D clinostats rotate the sample on a plane perpendicular to the rotation axis. These clinostats come in varying rotation speed and vessel body sizes. Rotating wall vessels (RWVs) are 2D clinostats with a larger body (5–20 cm in diameter) and a slower rotation speed (10–20 rpm). The culture media rotates at the same angular velocity as the rotating vessel wall, creating laminar fluid flow that minimises shear stress. The rotation frequency also prevents particle sedimentation, such that the cells remain at the centre of the vessel in near zero gravity.

#### 3D Clinostat – RPM

Random positioning machines (RPMs) consist of a sample area mounted at the centre of two frames that rotate independently with randomised speed and direction. The sample is therefore constantly reoriented randomly such that the gravity vector is averaged to near-zero. However, this only applies to the intersection of the two rotational axes. Accelerative forces from the rotation become stronger the further samples stray from centre, hence attention to sample positioning is critical on the RPM. Moreover, it has been shown that the RPM can induce stress responses in gravity-sensitive cell systems from its small vibrations and shear forces^59,60^. This can lead to cell detachment, and promote the formation of multicellular spheroid structures from increased intercellular interaction due to gravitational unloading^61^. Although, these effects were also observed in other clinostat types during multiple comparisons of 2D and 3D clinostats^62–64^. As such, studies involving either type of clinostat are widely accepted as models of microgravity.

### Human bone loss induced by simulated microgravity

So far, bone loss experiments under simulated-µG largely confirm the data collected in space. Bone resorption symptomatic of long-duration space missions is widely reproduced in bed rest studies on Earth^65–71^ (Table 3). Following 4, 14, or 30 days of 6° HDT, bone resorption is increased in test subjects as reflected by an increase in bone resorption markers, as well as an overall calcium imbalance^72,73^. Interestingly, bone resorption levels fail to be rescued despite increasing dietary calcium intake^74^. As previously mentioned, ALP is responsible for bone mineralisation. Consistent with the reportedly unchanged or decreased bone formation in space travellers^30,32,33^, levels of ALP remain constant^72^ or even decreased^73^ in HDT test subjects. Together, overall bone loss and alterations in biomarkers of bone turnover induced by simulated µG appears consistent with spaceflight data.

**Table 3:** Studies of bone-loss in humans subjected to simulated microgravity.

Technique	Duration (days)	Sample size	µG-related observations	Reference
HDT	30	12	• Increased markers of bone resorption by 20% and urinary calcium • Markers of bone formation, such as ALP remain unchanged	Morgan et al.^72^
HBR	6, 14	8, 9	• Serum calcium levels and ALP unchanged • Urinary calcium excretion greater in 14 days compared to 6 days bed rest • Increased bone resorption despite increased dietary calcium	Baecker et al.^74^
HDT	60	24	• Cortical bone density and thickness increased at non-weight-bearing distal radius, but trabecular density decreased • Trabecular density increased at weight-bearing distal tibia, but decreased in cortical compartment • Differential effects in different bones and bone compartments	Belavy et al.^75^
HBR	90, 56, 35, 24	8, 10, 10, 8	• Bone-loss more pronounced in trabecular compared to cortical compartment • Continued bone-loss after initial days of re-ambulation, and more cortical bone lost during this time	Cervinka et al.^77^
HBR / HDT	6	8	• Increased urinary calcium excretion and bone resorption markers • Osteoclast activity increased following 24 h of bed rest	Baecker et al.^65^ Heer et al.^66^
HBR	119	18	• Decreased BMD in spine, hip, calcaneus, pelvis and total body • Unchanged bone-specific ALP, decreased parathyroid hormone, but increased osteocalcin	Shackelford et al.^69^
HBR	30	8	• Increased markers of bone resorption and urinary calcium • Markers of bone formation unchanged	Smith et al.^70^
HDT	90	9	• Decrease in proximal femoral BMD • Increased bone resorption markers and urinary calcium • Resistive exercise increased bone formation but did not reduce bone resorption	Watanabe et al.^71^
HDT	21	15	• Decreased bone ALP and total ALP • Artificial gravity treatment by centrifugation failed to prevent BMD changes	Smith et al.^73^
HDT	60	8	• Reduced bone density in distal tibia and trabecular distal radius • Cortical thickness decreased at distal tibia but not distal radius • Exercise and nutrition countermeasures failed to prevent BMD changes	Armbrecht et al.^78^
HBR	56	10	• BMC loss in distal tibia epiphysis, but less severe in those subjected to resistive exercises • Most BMC loss recovered by 12-month follow-up	Rittweger et al.^79^
HDT	90	9	• BMC loss in tibia, but prevented in groups subjected to flywheel resistive exercise or pamidronate treatment	Rittweger et al.^150^
HBR	30	7	• Increased bone resorption markers and urinary calcium • Lower body negative pressure reduced BMD loss	Zwart et al.^151^
HDT	60	8	• Increased bone resorption markers • Resistive and aerobic exercise improved bone formation markers compared to controls • Exercise mitigated BMD loss in hip and leg	Smith et al.^73^

Summary of bone-related observations in humans from various microgravity-simulation studies.

*HDT* head-down tilt, *HBR* horizontal bed rest, *ALP* alkaline phosphatase, *BMD* bone mineral density, *BMC* bone mineral content.

On the other hand, there are some discrepancies in current literature describing how simulated- and true- µG affects different bone types and their compartments. One on-ground study subjected participants to a 60-day bed rest with 6° HDT, simulating the inactivity and fluid shift experienced in µG. The cortical area, thickness and density of the distal tibia in test subjects is decreased^75^, supporting the theory that µG negatively affects load-bearing bones more than non-weight-bearing bones^8,27–29^. In contrast to existing spaceflight data that indicates µG has little/no effect on non-weight-bearing bones^8,27–29^, HDT causes an increase in cortical area, thickness and bone density of the distal radius in subjects, despite a reduction in the trabecular area^75^. It is possible that an increased use of hands during the HDT study could be responsible for alterations in bone loading patterns, and is reflected in bone growth of the non-load-bearing distal radius. However, a meta-analysis of homogenous bone-related datasets from Gemini, Apollo, Soyuz, Skylab, Salyut, STS, Mir and ISS missions suggests a trend of underreporting positive bone density changes in the upper-limb and thorax region^76^. Taking into account data from both spaceflight and simulated-µG, the relationship between µG and the weight-bearing nature of bone or its compartments remains unclear. This calls for further clarification in future investigations.

Some advantages of studying bone-loss recovery in simulated-µG over spaceflight observations are that ground-based alternatives offer more accessible measurement timepoints, testing options, and longer post-µG-exposure monitoring. As such, they provide further insights into the limited understanding of the recovery process post-landing obtained from crewmembers alone. Consistent with post-landing data regarding the distal tibia, its cortical compartment generally recovers by 1 year following exposure to simulated-µG^75,77,78^. The recovery time of the tibia cortical thickness appears to be shorter in females (90 days)^78^ compared to males (180 days)^75^, despite both cohorts having been subjected to 60 days of 6° HDT. A combination of bed rest and unilateral lower limb suspension (ULLS) studies indicate that both cortical and trabecular compartments of the tibia suffer initial deterioration for the first month post-reambulation, with the cortical compartment to a larger extent^77^. However, unlike the cortical area of the tibia, the trabecular compartment does not recover even after 1–2 years post-ambulation^75,77,78^. Although the trabecular compartment of the tibia appears to begin recovering 3-6 months post-HDT^75,79^, this is followed by a reversal in BMD to −2% from 6 to 12 months in females^78^ and −1% from 3 to 24 months in males^75^. It is unlikely that these observations are related to age or biological sex, as biological sex of the two cohorts are balanced (*N* = 24 males and *N* = 24 females) with a similar age bracket (20- to 45-year-old males and 25- to 40-year-old females). It should be noted that similar to spaceflight data, recovery of non-weight-bearing bones such as the distal radius following exposure to simulated-µG is relatively underreported compared to weight-bearing-bones such as the hip and the distal tibia. Existing datasets can be reanalysed for radial bone data, and future studies can also direct more focus on the recovery of non-weight-bearing bones.

## Using animal and cell models to understand microgravity-induced bone loss

The establishment of cellular and animal models have greatly improved our understanding of the molecular mechanisms behind µG-induced bone loss. The following sections review our knowledge to date, comparing cell and animal data between spaceflight (Table 4) and simulated-µG (Table 5) experiments.

**Table 4:** Spaceflight studies of animals and bone cells.

Species	Cell type	Duration (days)	µG-related observations	Reference
Mice	N/A	8	• Bone volume decreased by 6.3% and bone thickness by 11.9% compared to GC • Increased active osteoclasts by 170% compared to GC • Increased osteocyte apoptosis by larger lacunar diameters	Blaber et al.^38^
Mice	N/A	30	• Femoral trabecular bone volume decreased 64% during spaceflight compared to GC • Increased bone resorption by 140% compared to GC • Osteocyte apoptosis reflected in reduced osteocyte lacunar volumes and increased lacunar vacancies	Gerbaix et al.^40^
Mice	N/A	33	• Spaceflight reduced BMD of whole body, and left and right femur by 8%, 11% and 8%, respectively compared to GC • Inhibition of myostatin/activin A signalling increases BMD in spaceflight mice comparable to untreated GC mice	Lee et al.^41^
Rat	N/A	14	• Reduced periosteal bone formation and collagen subunit mRNA levels in spaceflight rats compared to GC • Increased bone resorption during spaceflight, but with stable bone formation and matrix proteins expression • Oestrogen replacement partially rescued bone loss in spaceflight	Cavolina et al.^152^
Rat	N/A	14	• Spaceflight affect specific bones and bone compartments, but not strictly related to their weight-bearing nature • Reduced cortical femur, but not cortical humerus – both of which are weight-bearing	Keune et al.^42^
Rat	N/A	7	• Tibial trabecular bone volume reduced by 47–55%, thickness by 20–24% and density by 40–43% compared to GC	Vico et al.^36^
Monkey	N/A	14	• Young osteocytes in iliac crest show activated collagen protein biosynthesis for adaptive bone remodelling • Osteolytic activity of mature osteocytes intensified, leading to osteocyte destruction and increased empty lacunae compared to GC	Rodionova et al.^131^
Medaka fish	N/A	8	• Enhanced osteocalcin/osteorix in osteoblasts during spaceflight • Upregulated osteoclast activity during spaceflight – increased expression of TRAP, cathepsin K and MMP-9	Chatani et al.^153^
Human	BDSC	3	• Decreased expression of osteogenic differentiation markers Sox2, Oct3/4, Nanog and E-cadherin • Rapamycin induced transcriptional activation towards osteogenic differentiation	Gambacurta et al.^143^
Human	BMSC	14	• Cell cycle arrested after initial osteoblastic differentiation • Normal terminal differentiation to osteocyte inhibited	Bradamante et al.^86^
Human	Osteoblast	2.88	• Decreased focal adhesion contacts and F-actin fibre numbers • Counteracted by abrogating Rac1 and/or Cdc42	Guignandon et al.^84^
Mouse	Osteoclasts Preosteoclast	10	• Increased gene expression involved in osteoclast activation and function • Osteoclast bone resorption increased – increases in collagen telopeptide production compared to GC	Tamma et al.^116^
Mouse	Osteoblasts Osteoclasts	5	• Osteoblast have shorter and curvier microtubules, reduced number and size of focal adhesions, more condensed and fragmented nuclei compared to GC • Osteoblast cytoskeleton integrity affected • Increased osteoclast resorptive activity compared to GC	Nabavi et al.^83^
Mouse	Preosteoblast	4	• Reduced cytoskeletal stress fibres • Nuclei reduced in size by 30%, oblong shaped and fewer punctate areas • Reduced cell numbers by growth, but stable viability	Hughes-Fulford et al.^82^
Mouse	Osteoblasts	6, 42	• Trabecular osteoblasts more vulnerable to effects of µG compared to calcarial osteoblasts • PTHrP had anti-apoptotic effect on trabecular osteoblasts	Camirand et al.^154^
Mouse	Osteoblasts Osteoclasts	14	• Reduced expression of transcription factors and proteins for osteoblast differentiation • Increased osteoclast differentiation gene Cathepsin K and osteoclast activity • Irisin treatment promotes osteoblast differentiation and activity	Colucci et al.^120^
Chicken	Osteoblasts	3, 5	• Reduced collagen expression, leading to less extensive extracellular matrix • Reduced osteocalcin compared to GC	Landis et al.^87^
Goldfish	Scales	3.58	• Increased osteoclast activity • Increased osteoclast size and number of nuclei in multinucleated osteoclasts • Melatonin treatment reduced osteoclastic activation by increasing *Calcitonin* (osteoclast inhibitor) and decreasing *RANKL* (osteoclast promoter) mRNA expression	Ikegame et al.^117^
Goldfish	Scales	3.58	• Increased sclerostin production in osteoblasts • Suggest that sclerostin inhibits bone formation and activates osteoclasts	Yamamoto et al.^118^
Mice	Osteoblasts	14	• Irisin prevented µG-induced decrease in mRNA levels of *Runx2* and *Osterix*, and protein expression of collagen I and osteoprotegerin • Irisin could not prevent Trap and cathepsin K mRNA increased • Irisin could prevent osteoclastogenesis in µG by supporting osteoblast differentiation	Colucci et al.^120^

Summary of observations in animals and bone cells from various studies during spaceflights compared to respective ground controls.

*GC* ground control, *TRAP* tartrate-resistant acid phosphatase, *MMP-9* matrix metallopeptidase 9, *BDSC* blood-derived stem cells, *BMSC* bone marrow stem cells, *Sox2* sex determining region Y-box 2, *Rac1* Ras-related C3 botulinum toxin substrate 1, *Cdc42* cell division control protein 42 homolog, *µG* microgravity, *PTHrP* parathyroid hormone-related protein, *RANKL* receptor activator of nuclear factor κB ligand.

**Table 5:** Studies of bone loss in animal and cellular models subjected to simulated microgravity.

Cell type/species	Technique	Duration (days)	µG-related observations	Refs.
Preosteoblasts/Mice	HLU	28	• IL-6 expression increased in both sera and femurs of mice • IL-6-neutralising treatment alleviated bone loss reflected by increased BMD of tibia, trabecular thickness and number, bone volume fraction and load and stiffness of femur • IL-6 treatment increased mRNA expression of *ALP*, *osteopontin*, *Runx2* and decreased NFkB ligand protein in MC3T3-E1 cells	He et al.^103^
Osteoclast/mice	HLU	28	• Decreased femur BMD • Increased stimulation of osteoclastogenesis • Increased RANKL-stimulated osteoclastogenesis from precursors removed from tibia	Saxena et al.^126^
Osteoclast/mice	HLU	18	• Increased osteoclast numbers and resorptive activity following osteocyte apoptosis • Decreased bone density and compressive resistance	Aguirre et al.^127^
Osteoblasts/mice	HLU	14	• Reduced bone formation and osteocyte/osteoblast viability from decreased Wnt/β-catenin signalling • Increased sclerostin production, which inhibits bone growth by antagonising Wnt/β-catenin signalling	Lin et al.^107^
Preosteoblasts/rat	HLU Clinostat	28	• Increased bone loss in femurs, with decreased expression of transcription factors critical to osteoblast differentiation and increased mRNA expression of apoptotic proteins • Decreased cell activity and increased apoptosis in MC3T3-E1 cells	Dong et al.^100^
Preosteoblast/rat	HLU RWV	42	• Reduced BMD, trabecular thickness, trabecular number, ultimate load and stiffness in tibiae • Enhanced IL-6 in sera, skeletal muscle and tibiae • Hydrogen sulfide donor (GYY4137) treatment preserved bone structure in rats • GYY4137 stimulated expression of genes for osteoblastic differentiation and activity in MC3T3-E1 cells	Yang et al.^155^
MSC/mice	HLU	28	• Decreased osteogenic potential with reduced Runx2 expression • Enhanced adipogenic potential with increased PPARγ expression	Pan et al.^105^
Mice	HLU	28	• Elevated glucocorticoid signalling in osteoblasts, leading to cortical tibia bone loss • Osteoblast activity and bone formation inhibited • Osteoclast activity and bone resorption promoted • Increased sclerostin and RANKL-positive osteocytes, and apoptotic osteoblasts and osteocytes • Blocking glucocorticoid signalling prevents osteoblast cell death	Yang et al.^156^
Mice	HLU	28	• Reduced trabecular bone volume, surface area of cortical bone, maximum load and stiffness in tibia • Treatment with alendronate and anti-RANKL antibody inhibited bone resorption and restored bone mass close to control • Treatment with bortezomib increased whole bone mass by inhibiting bone resorption and promoting bone formation	Ding et al.^157^
Rat	HLU	14	• 66% increase in percentage of apoptosis in osteocytes • 14% increase in osteoclast number • 48% decrease in bone volume • Reloading returned apoptotic osteocytes and bone volume to baseline	Basso et al.^132^
Mice	HLU	3	• Increased osteocyte apoptosis in both trabecular and cortical bone, sequestered in endosteal cortical bone • Increased osteoclast number and cortical porosity • Decreased spinal BMD and vertebral strength	Aguirre et al.^127^
Rat	HLU	28	• Metaphyseal bone density reduced in hindlimb, but not in the proximal humerus • Opposite response of osteocyte proteins and osteoblast surface in hindlimb and forelimb bones within the same unloaded rat	Metzger et al.^136^
Mice	HLU	14	• Decreased Wnt/β-catenin signalling and upregulated Sost expression • Sclerostin suppressed osteoblast activity and viability of osteoblasts and osteocytes • Sost-ablated mice were resistant to HLU-induced bone loss and Wnt/β-catenin signalling was unaffected	Lin et al.^107^
Mice	HLU	7	• Osteocyte-ablated mice (with 20–30% remaining osteocytes) had fragile bone, osteoblastic dysfunction, and trabecular bone loss with microstructural deterioration • “Osteocyte-less” mice were resistant to HLU-induced bone loss	Tatsumi et al.^142^
Mice	HLU	28	• Preventative irisin treatment during unloading prevented bone loss in hindlimb • Irisin treatment following bone loss induced recovery of bone mass	Colaianni et al.^147^
Rat	HLU	28	• Decreased cancellous bone volume, higher osteoclast surfaces and lower bone formation rate in hindlimb and 4th lumbar vertebrae • Higher bone formation rate and lower osteoclast surfaces in proximal humerus • Osteocyte RANKL and sclerostin elevated in distal femur, but lowered in proximal humerus • Irisin treatment increased bone formation rate, lowered osteoclast surfaces and osteocyte RANKL and sclerostin	Metzger et al.^130^
Osteosarcoma/human	Clinostat	2	• Microgravity inhibited Runx2 activity and its responsiveness to BMP2 • Linked to actin microfilament disruption	Dai et al.^158^
Osteoblasts/human	RPM	1, 4.58	• Osteoblasts dedifferentiated assuming a spindle-shape and had decreased production of mineralisation crystals • Osteoblastic differentiation markers ALP, Runx2, BMP2 downregulated	Gioia et al.^94^
MSC/human	RCC	7	• Inhibition of osteogenic markers: ALP, collagen type 1, osteocalcin and Runx2 • Enhanced expression of adipogenic markers: adipsin, leptin, glut4 and PPARγ	Saxena et al.^159^
BMSC/human	RPM	4, 10	• Induced overexpression of Runx2, osterix, osteopontin and osteocalcin in non-osteogenic media • COL1A1 was upregulated, but only in the presence of osteogenic media	Cazzaniga et al.^115^
Osteoblasts/human	RPM	7, 14	• Cytoskeletal changes resulted in some cells detaching from the culture surface and forming multicellular spheroids • Increased expression of Sox9 and osteopontin after 7 and 14 days • Increased expression of osteocalcin and collagen type 1 after 14 days	Mann et al.^108^
Osteoblasts/human	Clinostat	20	• Inhibited calcium deposition with a complete absence of bone nodules compared to ground control • Cytoskeleton disruption and cells taking on a bulging morphology • Osteoblast inhibition in microgravity linked to repression of p38 phosphorylation	Yuge et al.^106^
Osteoblasts/mice	Clinostat	1	• Arrest of osteoblast cell cycle in the G2 phase due to a decrease in cyclin B1 expression associated with miRNA (specifically miR-181c-5p) inhibitory activity	Sun et al.^102^
Preosteoblasts/mice	RPM	1	• Inhibition of ALP, Runx2, osteocalcin, type 1 collagen and BMP expression • No changes in cell morphology	Hu et al.^96^
Preosteoblasts/mice	RPM	1	• Downregulation of ALP, osteocalcin, COL1A1, DMP1 and Runx2 gene expression	Hu et al.^160^
Osteoblasts/mice	RWV	1	• Decreased ALP, osteocalcin, AP-1 and Runx2 expression	Ontiveros and McCabe^97^
Preosteoblasts/mice	RWV	3	• Decreased ALP activity and inhibited RUNX2, BMP4, PthR1 and osteomodulin gene expression	Patel et al.^18^
Preosteoblasts/mice	RPM	3-9	• Inhibition of ALP activity and downregulated ALP, RUNX2, osteomodulin, PthR1 gene expression • Upregulation of Cathepsin K	Pardo et al.^99^
Osteoblasts Osteoclasts/mice	RPM	1	• Enhanced osteoclastogenesis by decreasing osteoblast production of OPG (increasing RANKL/OPG ratios)	Rucci et al.^121^
Preosteoblasts/mice	RWV	1	• Increased osteoclastogenesis and upregulated production/expression of factors involved in osteoclastogenesis e.g. cytokines, growth factors, proteases, signalling proteins and transcription factors c-Jun, MITF and CREB compared to ground control	Sambandam et al.^122^
Preosteoblasts Preosteoclasts/mice	RPM	7	• Inhibited expression of Runx2, Osterix, type I collagen α1 chain, RANKL and OPG genes in MCT3T3-E1 cells, which prevents osteoblast differentiation • Suppressed RANKL-dependent maturation of preosteoclasts	Makihira et al.^101^
Osteocyte / Mice	RWV	3	• Increased expression of *SOST*, sclerostin and *RANK/OPG* ratio	Spatz et al.^135^
Osteocyte-like/immortalised	RWV	5	• Disassembly of F-actin filaments and short dendritic processes at cell periphery • Increased Wnt1 and Sost expression • Reduced gene and protein level of β-catenin, with no nuclear translocation • Sclerostin antibody inhibited µG-induced down regulation of Wnt target genes and sclerostin protein expression	Yang et al.^140^

Summary bone-related observations in animal and cellular models from various microgravity-simulation studies.

*HLU* hindlimb unloading, *IL-6* interleukin 6, *BMD* bone mineral density, *ALP* alkaline phosphatase, *Runx2* Runt-related transcription factor 2, *NFB* nuclear factor kappa-light-chain-enhancer of activated B cells, *RANKL* receptor activator of nuclear factor kΒ ligand, *Wnt* wingless/integrated, *RWV* rotating wall vessel, *MSC* mesenchymal stem cell, *PPARγ* Peroxisome proliferator-activated receptor γ, *RPM* random positioning machine, *BMP2* bone morphogenic protein 2, *RCC* rotary cell culture, *COL1A1* Collagen type I alpha 1 chain, *Sox9* SRY-box transcription factor 9, *DMP1* dentin matrix acidic phosphoprotein 1, *AP-1* activator protein 1, *PthR1* parathyroid hormone 1 receptor, *OPG* osteoprotegerin, *MITF* melanocyte inducing transcription factor; *CREB* cAMP response element-binding protein, *SOST* gene encoding sclerostin.

### Osteoblasts

One of the most reported observations is changes in osteoblast cell morphology, which is reflected in actin-related cellular structures. Exposure to µG on a 4-day spaceflight causes collapse of the actin cytoskeleton in quiescent osteoblasts, with some cells exhibiting a spindle shape^80^, and some becoming rounder and covered with microvilli^81^. In addition, µG-exposure results in altered nuclei morphology; one study reports elongated nuclei with a 30% size reduction in spaceflight osteoblasts^82^, and another observes more condensed and fragmented nuclei compared to ground controls^83^. A symptom of altered actin cytoskeleton is the reduction in number of stress fibres and smaller stress fibre area in spaceflight osteoblasts^82,84^, indicating that µG impairs actin polymerisation. Focal adhesions are another mechanosensitive structure composed of polymerised actin, which appears to be destabilised upon exposure to µG. Consistent with the decrease in stress fibre formation, multiple studies observe reductions in the number of focal contacts and focal adhesion area in osteoblasts following spaceflight^80,83,84^. Moreover, focal contacts established during spaceflight appear less mature than those formed in ground control osteoblasts^83^, confirming that µG negatively impacts osteoblast adhesion.

Since cytoskeletal integrity is critical in signal transduction and expression of genes that regulate cell cycle, the loss of both actin cytoskeletal structure and cell adhesion can deter osteoblast proliferation in space. Indeed, µG delays cell cycle initiation in quiescent osteoblasts compared to ground-based analogues^82,85^. Human bone marrow stem cells (hBMSCs) aboard the ISS for 14 days encounter cell cycle arrest despite initial osteoblastic differentiation, and the phenotype of terminal differentiation to osteocyte is inhibited^86^. In support of this, spaceflight osteoblasts also demonstrate reduced osteocalcin and type I collagen expression, indicating reduced differentiation and matrix development^87^. However, it should be noted that despite the impairment of osteoblastic proliferation and differentiation under µG, genome-wide and Next Generation Sequencing analysis does not suggest apoptosis or cell senescence^86^. The authors hypothesised that BMSCs or immature osteoblasts respond to µG by reverting to a quiescent state, in line with the observations of increased BMSC differentiation potential seen in µG-exposed mice following reloading on Earth gravity^88,89^.

Congruent to spaceflight observations, most of the current literature consistently suggest that simulated-µG inhibits the proliferation and differentiation of MSC towards osteoblasts, despite using different methods of µG simulation (Table 5). This is indicated by the suppressed gene expression of osteoblast differentiation markers such as bone morphogenic protein (BMP) and Runt-related transcription factor 2 (Runx2)^18,90–101^, in addition to the lowered expression of other osteoblastogenesis-related genes^18,97,99,101–103^. The marker for osteoblast maturation from MSC progenitors – ALP, is also expressed at lower levels in preosteoblasts and osteoblasts exposed to various forms of simulated-µG^95–97,99,104^. Subsequently, enzymatic activity of ALP is reduced in these cells^18,99,103^. Indeed, MSCs extracted from femurs of rats subjected to HLU for 28 days show reduced Runx2 and ALP mRNA expression, as well as decreased ALP activity^105^. However, the authors did not mention whether these changes in biomarker expressions correlated to any physical BMD changes in the rats.

The reduced expression and activity of ALP in simulated-µG-cells correlates well with decreased secretion of matrix proteins such as type 1 collagen and osteocalcin^95–97,99^, which could lead to reduced production of mineralisation crystals^93,94^. It is suggested that simulated-µG prevents extracellular calcium from entering osteoblasts, thus reducing intracellular free calcium levels which impairs calcium deposition and bone formation^102,106^. Furthermore, simulated-µG has been shown to cause cell cycle arrest at G2 and even induce apoptosis in osteoblasts^100,102^. HLU-induced bone loss in mice is consistently accompanied by a reduction in the number of viable osteoblasts and osteocytes in mice^107^. It is of note that some studies observed morphological changes in osteoblasts treated with simulated-µG, where actin cytoskeleton disruptions cause “bulging” or “spheroidal” morphologies^94,106,108^. However, while Hu et al. have reported simulated-µG-induced inhibition on preosteoblast differentiation, no alterations in cell morphology is observed^96^. Of note, studies that report morphological changes subjected bone cells to extended periods of simulated-µG (up to 20 days)^94,106,108^, compared to the 7-day-exposure period by Hu et al.^96^. One study attributes the inhibition of osteogenesis in simulated-µG to the obliteration of primary cilia on osteoblasts cultured on the RPM^93^, suggesting that primary cilia play a sensory role in bone metabolism^109,110^. Since the actin cytoskeleton plays a critical role in osteogenic differentiation^111^ and cell cycle processes^112^, it is possible these phenomena could be symptoms of µG-induced disruptions. Collectively, these observations provide strong evidence that impaired differentiation, maturation and proliferation of osteoblasts could be responsible for the µG-induced reduction in bone formation^93,113^.

Conversely, a smaller fraction of the literature suggests an opposing argument, that simulated-µG in fact promotes proliferative and differentiation capabilities in MSCs^114^, and does not directly induce osteoblast cell death^104^. RPM-induced µG causes MSCs to express increased levels of Runx2 and Osterix (Osx)^115^, which are transcription factors essential in osteoblastic differentiation. Runx2 gene expression also remains unaltered in osteoblasts cultured on the RPM compared to 1 G controls^104^. Furthermore, osteoblasts exposed to simulated-µG show elevated mRNA expression of SOX9^108^, which is a transcription factor characteristic of commitment to the osteoblastic lineage from MSC differentiation. Consequently, simulated-µG reportedly increases bone matrix protein production in MSCs and osteoblasts/osteoblast-like cells, such as osteopontin and osteocalcin gene expression, and type 1 collagen secretion^104,108,115^. It should be noted that immortalised osteoblast/osteoblast-like cell lines are used in some of these studies, and the effects of inherent mutations must be considered. Nonetheless, further investigation is required to either confirm or dispute these observations in future studies.

### Osteoclasts

Mechanosensitive osteoclasts also contribute to space-related bone loss by disrupting normal bone homeostasis. Although much less studied than osteoblasts, osteoclasts have demonstrated increased resorptive activity in response to µG compared to controls on Earth^83,116–118^. Mature osteoclasts cultured on ivory or bovine bone slices demonstrate an increase in number of resorption pits formed following 5 or 7 days of spaceflight, respectively, compared to their relative ground controls^83,116^. Indeed, µG induced a dramatic increase in the expression of bone resorption-related genes, as well as elevated collagen telopeptide production^116^, which is correlated with bone resorption^119^. The increase in resorptive activity is reflected in the faster differentiation and maturation of spaceflight osteoclasts. Expression of genes involved in osteoclast differentiation, such as integrin β_3_, cathepsin K, MMP‐9 and calcitonin receptor are significantly upregulated following µG exposure in comparison to controls on Earth^116,117,120^. Collectively, these observations in spaceflight experiments indicate that osteoclasts play a critical role in µG-induced bone loss.

There are limited reports of osteoclast behaviour in simulated-µG, however, current literature is in consensus that simulated-µG promotes osteoclastogenesis and osteoclast function^121–127^. Osteoclastogenesis can be influenced by secretion of osteoprotegerin (OPG) and receptor activator of nuclear factor κB ligand (RANKL) from osteoblasts and osteocytes. *RANKL* – an osteoclastogenesis promotor, binds *receptor activator of nuclear factor κB (RANK)* receptors on the surface of osteoclasts for differentiation/maturation, while OPG acts as a decoy receptor that also binds *RANKL* – thus serving as a negative regulator of osteoclastogenesis^128,129^. RPM-facilitated µG decreases osteoblast production of OPG, thereby increasing RANKL/OPG ratios, which enhances osteoclastogenesis^121^. Supporting this observation, RANKL-stimulated osteoclastogenesis is increased in mice despite 28 days of HLU^126^. Autophagosome production reportedly enhances osteoclast differentiation, and RWV treatment enhances expression of autophagic genes^123^. This in turn promotes differentiation of osteoclasts, while inhibition of autophagy by 3-methyladenine conversely prevents osteoclastogenesis despite simulated-µG-exposure^123^. The positive effect of simulated-µG on osteoclastogenesis is also evidenced by the upregulation of cytokines, growth factors, proteases, signalling proteins and transcription factors such as c-Jun, MITF and CREB in osteoclasts cultured in RWV compared to 1 G controls^122^. Mice subjected to HLU displays increased osteoclast number, elevated osteoclast surfaces in hindlimb and vertebral sites, and ultimately reduced spinal BMD and strength^127,130^. µG-stimulated osteoclastogenesis and osteoclast activity are consistently observed in both on-ground and in-space experiments, highlighting the opportunity of these processes as a therapeutic target against bone loss.

### Osteocytes

Osteocytes reside in the cavities of the mineralised bone matrix, also known as lacunae (Fig. 1), where they synthesise proteins such as collagen and glycosaminoglycans which contributes to bone mineralisation^131^. µG-exposure from spaceflight is found to impede the differentiation of osteoblasts into osteocytes^131^. This causes underdevelopment in the Golgi complexes of osteocytes responsible for the secretion of matrix proteins, leading to retardation in bone matrix mineralisation^131^. Furthermore, osteocytes are reported to undergo apoptosis as early as three days into µG-exposure from spaceflight, leaving an increased number of empty lacunae^40,131^, or lacunae with reduced volume and altered shape^40^. On top of reduced bone formation/mineralisation, this may trigger further bone resorption to cause deterioration of bone microstructure and loss of bone mass^40^.

Congruent to spaceflight observations, HLU rats demonstrated an increase in apoptotic osteocytes by 66% compared controls^132^, while HLU mice displayed enhanced osteocyte apoptosis in their spine^127^. The death of osteocytes is associated with increased osteoclast activity^133,134^. Osteocytes can promote osteoclast-mediated bone loss by secreting proteins such as sclerostin – an inhibitor of bone formation, and RANKL – a promotor of osteoclastogenesis. Simulated µG exposure increases RANKL and sclerostin gene expression in osteocytes cultured in RWV^135^, suggesting that osteocytes play a role in µG-induced bone resorption. The mechano-sensitive influence of osteocytes on osteoclasts can be demonstrated even within the same animal in a rodent HLU model. Osteocyte proteins (including sclerostin) is elevated in the unloaded femur compared to the weight-bearing forelimb of the same HLU rat, which coincides with decreased osteocyte number and increased osteoclast activity of the unloaded hindlimb compared to the loaded forelimb^136^.

The elevated sclerostin secretion by osteocytes also negatively regulates oasteoblast-mediated bone formation by antagonising BMP/Wnt signalling^137–139^. Simulated µG has been shown to depress the Wnt signalling pathway and downregulate cell cycle related genes such as Cyclin D1 in osteocyte-like cells, which could be partially restored by the administration of antibodies against sclerostin^140^. Indeed, the ablation of sclerostin or RANKL appears to be protective over unloading-induced bone loss in HLU mice models^107,141^. Interestingly, osteocyte-deficient mice demonstrated resistance to HLU-induced bone loss, where bone loss and microstructural deterioration is prevented despite unloading^142^. Thus, osteocytes appear to play a vital role in promoting osteoclastogenesis and bone resorption upon mechanical unloading or µG-exposure. Future studies should leverage the unique ability of osteocytes to influence both osteoblasts and osteoclasts when exploring therapeutic interventions against bone loss.

Despite being the most abundant type of bone cells, osteocytes represent the least studied cell type due to their inaccessibility from being embedded in the bone matrix. The current literature landscape also points to a general lack of methods for osteocyte isolation and culture. In addition, isolated osteocytes may not be physiologically relevant to osteocytes in vivo, as their native environment is three dimensional and complex. As such, there is a need to establish more robust protocols for osteocyte culture and characterisation.

### Molecular therapies against microgravity-induced bone loss

Research using cell and animal models of µG-induced bone loss has revealed various possible therapeutic targets as described above, while providing accessible and ethical platforms for the testing of molecular therapeutic agents. Multiple agents have been identified to prevent µG-induced bone loss by promoting osteoblast differentiation, maturation and activity. Rapamycin treatment of human blood-derived stem cells (hBDSCs) aboard the ISS for 3 days induces earlier differentiation towards osteogenic lineage cells compared to analogous ground controls in 1G^143^. hBDSCs exposed to µG exhibit reduced expression of embryonic markers Sox2, Oct3/4, Nanog and E-cadherin to a larger extent compared to ground controls, indicating that rapamycin induces an earlier loss of pluripotency in space than on Earth^143^. Furthermore, expression of differentiation-related transcription factors in µG-exposed hBDSCs are altered in favour towards osteogenesis^143^. Downregulation of the transcription factors Otx2 and Snail in µG inhibits osteogenic differentiation; while GATA4 and SOX17, which promote differentiation towards osteogenesis are upregulated^143^. In another study, IL-6 neutralisation prevented the reduction in Runx2 and ALP mRNA expression, as well as ALP activity induced by simulated-µG treatment^103^. Similarly, IL-6-neutralisation successfully alleviated HLU-induced bone loss in mice tibia^103^. Irisin administration has also been shown to prevent µG-induced downregulation of transcription factors and proteins critical to osteoblast differentiation and activity^120^. Similar to IL6-neutralisation, irisin treatment elevated Runx2 and ALP expression, and ALP activity, as well as increased osteoblast numbers and bone formation in mice^144–146^. These observations suggest that the administration of rapamycin, irisin and IL-6 neutralisation can prevent µG-induced downregulation of osteoblast differentiation, maturation and mineralisation activity.

Irisin has been demonstrated to prevent bone loss induced by HLU in mice^147^. As previously discussed, osteocytes can secrete sclerostin to promote osteoclast-mediated bone loss. The number of sclerostin-positive osteocytes is higher in the hindlimb of HLU mice compared to controls^130^. Upon irisin treatment, the sclerostin-positive population was significantly lowered, indicating a negative effect on osteoclast activity as an increase in bone formation rate was observed^130^. Irisin has also been demonstrated to downregulate osteoclastogenesis. Its administration reduced RANKL-induced osteoclastogenesis^146^, and decreased osteoclast-covered bone surfaces in HLU mice compared to ambulatory controls^130^. Analogously, melatonin treatment upregulated osteoclast inhibitor *calcitonin* and downregulated *RANKL* in goldfish scales^117^. However, it is unknown whether this translates into suppression of osteoclast activity in this study. Nevertheless, these molecular compounds all serve as promising therapeutic agents against µG-induced bone loss. However, further animal studies will be required prior to phase 1 human clinical safety trials.

## Conclusion

Through the combination of spaceflight and simulation data on humans, animals and cellular models, we are continuously improving our knowledge of µG-induced bone loss. Our current understanding suggests µG affects weight-bearing bones to a larger degree than non-weight-bearing bones. However, this relationship warrants further investigation, especially with the underreporting of changes in non-weight-bearing bones in astronauts. Moreover, there are inherent biases in HDT/HBR studies, where the use of non-weight-bearing bones, such as the distal radius, may be increased in subjects. Together, these observations might mask the true effect of µG on non-weight-bearing bones. Future studies can also clarify the discrepancies in the effect of µG on different bone compartments, as well as how µG influences recovery processes.

The use of cellular models has shed light into the molecular mechanisms behind µG-induced bone loss, which in turn provides potential targets for therapeutic intervention. Although bone loss stems from both reduced bone formation by osteoblasts and elevated resorption by osteoclasts, the current literature landscape is largely focused on osteoblasts. Preliminary observations of increased osteoclast activity in µG present promising therapeutic targets, as such, the role of osteoclasts in bone loss requires further elucidation. In addition, as osteocytes are capable of influencing both osteoblasts and osteoclasts, this unique position should be leveraged for potential therapeutic options. Thus, the role of osteocytes in balancing bone resorption and formation under µG also deserves further clarification. Collectively, these efforts should enable the development of more effective preventative and therapeutic measures against µG-induced bone loss, thus paving the way for safer journeys as we venture further away from Earth.

### Future directions

- Extend monitoring of astronauts post-mission for at least 12 months to investigate the possibility of progressive fragility, particularly in non-weight-bearing bones such as the distal radius.
- Clarify the relationship between µG and the weight-bearing nature of bone loss, particularly whether µG affects non-weight-bearing bones.
- Clarify how µG affects different bone compartments in humans and animals.
- Investigate how the bone-loss recovery process differs between weight-bearing and non-weight-bearing bones, as well as between different bone compartments.
- Address discrepancies in the literature that points to µG promoting osteogenic proliferation and differentiation.
- Establish robust differentiation, isolation and culturing protocols for osteoclasts and osteocytes, as well as characterisation and validation of existing osteoclast/osteocyte-like cell lines.
- Dissect the mechanisms behind how osteoclasts and osteocytes contribute to µG-induced bone loss to identify possible therapeutic targets.

### Reporting summary

Further information on research design is available in the Nature Research Reporting Summary linked to this article.

## Supplementary information

Reporting Summary

## Data Availability

No datasets were analyzed or generated in the writing of this review.

## Code availability

No codes were used in the writing of this review.
